# Oncogene addiction to c-MYC in myeloma cells

**DOI:** 10.18632/oncotarget.631

**Published:** 2012-08-25

**Authors:** Toril Holien, Anders Sundan

**Affiliations:** KG Jebsen Center for Myeloma Research and Department of Cancer Research an Molecular Medicine, Norwegian University of Science and Technology, Trondheim, Norway; KG Jebsen Center for Myeloma Research and Department of Cancer Research an Molecular Medicine, Norwegian University of Science and Technology, Trondheim, Norway

Analysis of DNA from primary cancer cells has revealed large numbers of genetical aberrations, raising questions as to which mutations are “drivers” and which are “passengers”. Applying a small molecular inhibitor of MYC-MAX heterodimerization [1] we recently reported that myeloma cells treated with inhibitor rapidly undergo apoptosis [2]. Thus, a large fraction of primary myeloma cells apparently is dependent on c-MYC activity for survival.

We came to these findings by studying an entirely different aspect of myeloma cell biology, namely the mechanisms behind bone morphogenetic protein (BMP)-induced apoptosis of myeloma cells. It has been known for several years that dependent on receptor expression, various BMPs may potently induce myeloma cell death, suggesting a role for BMPs in suppressing myeloma development [3]. Analyzing the early phases of BMP-induced apoptosis in a myeloma cell line we found that the majority of genes differentially expressed between cells going to die and surviving cells also were known as transcriptional targets for c-MYC [4]. Furthermore, we could show that BMP-induced apoptosis correlated with c-MYC protein downregulation, and that c-MYC expression from a strong viral promoter protected cells from BMP-induced apoptosis. Even more interesting was a close examination of primary myeloma cells from fifteen patients where both BMP signaling and c-MYC protein status could be analyzed. Besides from expressing c-MYC protein, the cells from these patients fell into three categories; the large majority of patients (11 out of 15) had cells where BMP-induced apoptosis correlated with c-MYC downregulation. Two of the patients had cells with proper BMP-signaling but without effects on c-MYC expression levels and cell viability. These cells had translocations placing *MYC* under control of an immunoglobulin enhancer, thereby apparently overriding the BMP signal. The last two patients had cells with constitutive BMP signaling and no effects of added BMPs, suggesting that malignant cells from these patients had adapted to life in the presence of active BMP signaling.

Taken together, the results indicate that BMP-induced apoptosis in myeloma cells is dependent on downregulation of c-MYC. However, they also suggested that if we could inhibit the activity of c-MYC in myeloma cells by other means, a majority of myeloma cell clones would not survive.

A role for c-MYC in myeloma cells has earlier been suggested by gene expression studies indicating that the transcriptional signature of c-MYC could be detected in approximately 70% of primary myeloma clones in contrast to cells from the pre-malignant condition, monoclonal gammopathy of undetermined significance (MGUS) [5]. Furthermore, c-MYC has been shown to be important for survival of cell lines because downregulation of c-MYC by RNA interference induces apoptosis in some myeloma cell lines [6]. However, a major difference between myeloma cell lines and primary cells is that the latter proliferate very slowly, and as MYC also has been implicated in cell proliferation, it was not obvious what the role for c-MYC was in primary myeloma cells.

Based on this we went on to study the effects on myeloma cells applying an inhibitor of MYC-MAX heterodimerization; the 10058-F4 compound. Treatment with 10058-F4 led to induction of apoptosis in primary myeloma clones, indicating that a majority of these cells were dependent on c-MYC activity for survival. Genetic rearrangements affecting MYC are considered late events in myeloma development and are common in myeloma cell lines that are derived from late stage plasmacytomas [7]. As the cells analyzed in our studies were obtained from newly diagnosed, untreated patients, the results also suggest that c-MYC deregulation may be a relatively early event in the course of multiple myeloma.

The study raises other questions. First of all, the specificity of small molecular pharmacological inhibitors is always a matter of concern. Moreover, the 10058-F4 compound represents a new class of protein inhibitors because it does not inhibit enzyme activity but rather the bi-molecular interaction between c-MYC and its partner MAX. However, we found that the 10058-F4 compound did not affect survival of the U266 myeloma cell line even at high concentrations, and the U266 cell line is clearly independent of c-MYC because it does not express c-MYC. Secondly, it is not obvious that results from *in vitro* studies are relevant *in vivo*. On the other hand we could show that the 10058-F4 compound also killed myeloma cells in the presence of bone marrow stromal cells which mimic some aspects of the bone marrow microenvironment.

Despite significant advances in treatment during the last couple of decades, multiple myeloma remains incurable and better therapies are needed. The efficacy of MYC inhibition in therapy remains, however, to be determined. Unfortunately, the 10058-F4 compound is not applicable *in vivo* due to its rapid degradation [8], so better drugs are needed. However, there is great hope for this approach to treatment because animal experiments have shown that lymphomas recurring after MYC suppression continued to exhibit oncogene addiction to c-MYC [9]. c-MYC is in many cases central to cancer cell survival and due to its functional nonredundancy, it may not be easily replaced by alternative signaling mechanisms [10].
